# Six new species of *Arthrinium* from Europe and notes about *A.caricicola* and other species found in *Carex* spp. hosts

**DOI:** 10.3897/mycokeys.49.32115

**Published:** 2019-03-12

**Authors:** Ángel Pintos, Pablo Alvarado, Juan Planas, Rene Jarling

**Affiliations:** 1 Departamento de Investigación Mycologica, Cultivos Pima SL, Son Peretó 50 bajos, 07013 Palma de Mallorca, Spain Departamento de Investigación Mycologica Palma de Mallorca Spain; 2 ALVALAB, La Rochela 47, 39012 Santander, Spain ALVALAB Santander Spain; 3 Carrer can Socies 12, 07010 Palma de Mallorca, Spain Unaffiliated Palma de Mallorca Spain

**Keywords:** Apiosporaceae, Ascomycota, Sordariomycetes, Xylariales, ITS, 28S rDNA, tef1, tub2

## Abstract

Several new *Arthrinium* specimens were collected from various locations in Mediterranean and temperate Europe. A collection of the type species, *A.caricicola*, was obtained from dead leaves of *Carexericetorum* in Berlin. Sequences of four genetic markers, ITS, 28S rDNA, tef1 and tub2 were produced from almost all collections and analyzed with those available in public databases. Results are employed to support six new species: *A.balearicum*, *A.descalsii*, *A.esporlense*, *A.ibericum*, *A.italicum* and *A.piptatheri*. The type species, *A.caricicola*, is related to other species occurring on *Carex* sp.; these might represent an independent lineage from *Apiospora* and the remaining species of *Arthrinium*. Finally, the sexual morph of *A.marii* is described and illustrated for the first time.

## Introduction

The genus *Arthrinium* Kunze (Apiosporaceae, Sordariomycetes) differs from other anamorphic genera because of the presence of basauxic conidiophores, which arise from structures called conidiophore mother cells (Schmidt and Kunze 1817; Hughes 1953; Minter 1985). This infrequent type of conidiogenesis can be found also in *Cordella* Speg., *Dictyoarthrinium* S. Hughes, *Pteroconium* Sacc. ex Grove, and *Spegazzinia* Sacc. (Ellis 1971), but *Pteroconium* and *Cordella* are now considered synonyms of *Athrinium* (Seifert et al. 2011; Crous and Groenewald 2013). *Apiospora* Sacc., the sexual state of *Arthrinium*, is also considered a synonym based on the one fungus-one name policy (Hawksworth et al. 2011; Crous and Groenewald 2013), and *Nigrospora* Zimm. is thought to be the closest relative (Wang et al. 2017).

There are about 80 valid species names of *Arthrinium*. The most significant contributions to species diversity of *Arthrinium* before the DNA-era were those of Schmidt and Kunze (1817), Kunze and Schmidt (1823), Fuckel (1870, 1874), Ellis (1963, 1965, 1971, 1976), and Larrondo and Calvo (1990, 1992). Genetic evidence allowed to confirm some of these taxa and propose multiple new species, e.g. Crous and Groenewald (2013), Singh et al. (2013), Dai et al. (2016, 2017), Jiang et al. (2018), and Wang et al. (2018). Smith et al. (2003) produced the first genetic data (18S and 28S rDNA) of *A.phaeospermum* (Corda) M.B. Ellis, supporting that this genus, as well as *Apiospora*, represent a separate family within Xylariales. This was later confirmed by Spatafora et al. (2006) and Zhang et al. (2006) who added new information from gene-coding DNA markers (18S and 28S rDNA, tef1, rpb2). Singh et al. (2013) published a ITS rDNA phylogeny including several type sequences obtained by Ogawa et al. (unpublished), such as those of *A.marii* Larrondo & Calvo, *A.hispanicum* Larrondo & Calvo, *A.mediterranei* Larrondo & Calvo, *A.serenense* Larrondo & Calvo, and *A.phaeospermum*, and introduced the new species *A.rasikravindrae* Shiv M. Singh, L.S. Yadav, P.N. Singh, Rah. Sharma & S.K. Singh (as *rasikravindrii*). Soon afterwards, Crous and Groenewald (2013) published a comprehensive re-evaluation of *Arthrinium* based on multigenic data, introducing eight new species and providing genetic data from several type strains of other taxa. They formally proposed the synonymy between *Arthrinium* and *Apiospora*, giving priority to *Arthrinium*, but provided no data of the type species, *A.caricicola* Kunze & J.C. Schmidt. Sharma et al. (2014) published the new species *A.jatrophae* R. Sharma, G. Kulk. & Shouche and built a phylogenetic tree based on rDNA that showed three main clades: one formed by *A.urticae* M.B. Ellis, a second including *A.puccinioides* Kunze & J.C. Schmidt and *A.japonicum* Pollack & C.R. Benj., and a third including the remaining known species of *Arthrinium* and *Apiospora*. Multigenic data of the first two clades was first obtained by Ogawa et al. (unpublished), and also Crous and Groenewald (2013), although they did not include these data in their phylogenetic analyses. Some new species of *Arthrinium* were described in the next years (Crous et al. 2015; Senanayake et al. 2015; Hyde et al. 2016; Dai et al. 2016, 2017; Wang et al. 2018; Jiang et al. 2018), and the multilocus phylogenetic analysis revealed that the sister clade of *Arthrinium* was *Nigrospora* in Apiosporaceae (Wang et al. 2017).

Morphological features traditionally employed to discriminate between species of *Arthrinium* include conidial shape, conidiophores, presence or absence of sterile cells and the presence of setae. Two great groups of species can be discriminated: 1) those with irregularly shaped conidia (including the type species *A.cariciola* and several others mainly associated with *Carex* spp. (Cyperaceae, Poales), such as *A.austriacum* Petr., *A.fuckelii* Gjaerum, *A.globosum* Koskela, *A.japonicum*, *A.kamtschaticum* Tranzschel & Woron., *A.morthieri* Fuckel, *A.muelleri* M.B. Ellis, *A.naviculare* Rostr., *A.puccinioides* and *A.sporophleum* Kunze), and 2) the remaining species with globose to ellipsoid conidia, mainly associated with other plants in the Poales (Cyperaceae, Poaceae, Restionaceae), e.g. *A.pterospermum* (Cooke & Massee) Arx, *A.phragmitis* Crous, *A.sacchari* (Speg.) M.B. Ellis, *A.saccharicola* F. Stevens, *A.kogelbergense* Crous, and *A.hysterinum* (Sacc.) P.M. Kirk, or even a wider diversity of potential hosts, such as *A.arundinis* (Corda) Dyko & B. Sutton, *A.phaeospermum*, *A.rasikravindrae* and *A.malaysianum* Crous.

Spatafora et al. (2006) and Zhang et al. (2006) were the first to obtain genetic data from the type species of *Apiospora*, *Ap.montagnei* Sacc. (CBS 212.30, AFTOL-ID 951) and suggested that it belongs in a distinct family within Xylariales. Sequences of a few other species of *Apiospora* are also available, including *Ap.sinensis* K.D. Hyde, J. Fröhl. & Joanne E. Taylor (HKUCC 3143 in Smith et al. 2003), *Ap.setosa* Samuels, McKenzie & D.E. Buchanan (ICMP 6888 /ATCC 58184 ex type PDD 41017 in Huhndorf et al. 2004), and *Ap.tintinnabula* Samuels, McKenzie & D.E. Buchanan (ICMP 6889-96 ex type PDD 41022 in Jaklitsch and Voglmayr 2012). Jaklitsch and Voglmayr (2012) produced a 28S rDNA phylogeny where the type species *Ap.montagnei* seemed not significantly different from *Ap.sinensis* but distinct from the other species sequenced. In addition, some *Apiospora* sexual morphs have been biologically linked with putatively prioritary *Arthrinium* taxa: *A.hysterinum* = *Ap.bambusae* (Turconi) Sivan. (Sivanesan 1983; Kirk 1986; Réblová et al. 2016), *A.arundinis* = *Ap.montagnei* (Hyde et al. 1998), and *A.sinense* = *Ap.sinense* (Réblová et al. 2016). However, none of these putative synonymies has been confirmed with genetic data, as some type collections are missing or too old for standard DNA analysis.

The aim of the present study was to study new *Arthrinium* samples found in temperate and southern Europe, including one specimen of *A.caricicola* and several putatively new species, and compare them morphologically and genetically with existing taxa. In some cases, e.g. *Ap.tintinnabula*, type collections were loaned and additional sequences obtained to delimit the genetic boundaries of some species.

## Materials and methods

### Pure culture isolation

During the surveys conducted in 2017 and 2018, 34 fresh specimens were collected from various plant hosts in Germany, Italy, Portugal and Spain. To isolate the sexual morph, ascomata were removed from the stromata using a sterile razor blade, transferred to a water droplet mounted on a microscope slide, torn apart with forceps to release the ascospores from asci, and pipetted on a 2% malt extract agar (MEA) plate supplemented with 200 mg/L penicillin G and streptomycin sulphate. Germinated ascospores were then transferred to MEA 2% plates, which were sealed with plastic film and incubated at room temperature. To isolate the asexual morph, plate cultures were superficially scrapped with a needle to dislodge conidia that were transferred to a drop of water. The suspension was then picked up with a syringe, and small droplets sown on a MEA 2% plate supplemented with 200 mg/L penicillin G and streptomycin sulphate. The germinated conidia were then transferred to 2% MEA plates, which were sealed with laboratory film and incubated at room temperature. Cultures were deposited at CBS-KNAW Fungal Biodiversity Centre, Utrecht, The Netherlands (CBS).

### Morphological observations

Hand sections of stromata or conidiomata were made using a razor blade and mounted in water on a microscope slide. Observations were made with a Zeiss Axioscop microscope using differential interference contrast (DIC), images were taken with a FLIR camera with A. Coloma open source software. Measurements were taken with FIJI ImajeJ software, reported with maximum and minimum values in parentheses, and the range representing the mean plus and minus the standard deviation, followed by the number of measurements in parentheses. For certain images of conidiophores, the image stacking software Zerene Stacker v. 1.04 (Zerene Systems LLC, Richland, WA, USA) was used. Morphological descriptions were based on cultures sporulating on 2% MEA medium at room temperature. The original specimens were deposited at the fungarium of the Real Jardin Botanico de Madrid (MA-Fungi).

### DNA isolation, amplification and phylogenetic analyses

Total DNA was extracted from dry specimens employing a modified protocol based on Murray and Thompson (1980). PCR amplification was performed with the primers ITS1F and ITS4 (White et al. 1990; Gardes and Bruns 1993) for ITS region, while LR0R and LR5 (Vilgalys and Hester 1990; Cubeta et al. 1991) were used to amplify the 28S rDNA region, T1, Bt2a, and Bt2b (Glass and Donaldson 1995; O’Donnell and Cigelnik 1997) for the β-tubulin gene (tub2), and EF1-728F, EF1-983F and EF1-1567R (Rehner and Buckley 2005) for the translation elongation factor 1a (tef1) gene. PCR reactions were performed under a program consisting of a hot start at 95 °C for 5 min, followed by 35 cycles at 94 °C, 54 °C and 72 °C (45, 30 and 45 s respectively) and a final 72 °C step 10 min. PCR products were checked in 1% agarose gels, and positive reactions were sequenced with one or both PCR primers. Chromatograms were checked searching for putative reading errors, and these were corrected.

BLAST (Altschul et al. 1990) was used to select the most closely related sequences from INSDC public databases. Sequences came mainly from Crous and Groenewald (2013), Singh et al. (2013), Sharma et al. (2014), Crous et al. (2015), Senanayake et al. (2015), Dai et al. (2016, 2017), Hyde et al. (2016), Réblová et al. (2016), Jiang et al. (2018), and Wang et al. (2018), as well as Ogawa et al. (unpublished). Two distinct alignments were built in MEGA 5.0 (Tamura et al. 2011) and aligned with Clustal W with manual corrections: 1) a multigenic alignment including ITS, 28S rDNA, tub2 and tef1 data (without introns) from all Apiosporaceae and related families, and 2) a second alignment built with the same DNA markers (with introns) including only species related with *A.sacchari* (/saccharii clade). Introns were removed from tef1 and tub2, and GBlocks (Castresana 2000) was employed to remove 201 ambiguously aligned sites from ITS rDNA in the Apiosporaceae alignment, but not in the alignment of the /sacchari clade, in order to resolve this complex with all the phylogenetic signal available. The final alignment of the Apiosporaceae included five partitions with 217/461 (ITS rDNA), 229/846 (28S rDNA), 78/252 (tub2), 43/147 (tef1 EF1-728F to EF1-983F), and 76/413 (tef1 EF1-983F to EF1-1567R) variable sites, while the final alignment of the /sacchari clade had 35/535 (ITS rDNA), 18/837 (28S rDNA), 99/719 (tub2), 68/429 (tef1 EF1-728F to EF1-983F), and 4/407 (tef1 EF1-983F to EF1-1567R) variable sites. The aligned loci were loaded in PAUP* 4.0b10 (Swofford 2001) and subjected to MrModeltest 2.3 (Nylander 2004). Model GTR+G+I was selected and implemented in all partitions in MrBayes 3.2.6 (Ronquist and Huelsenbeck 2003), where a Bayesian analysis was performed (data partitioned, two simultaneous runs, six chains, temperature set to 0.2, sampling every 100^th^ generation) until convergence parameters were met after about 3.43M generations (Apiosporaceae) or 0.9M (/sacharii clade), standard deviation having fell below 0.01. Finally, a full search for the best-scoring maximum likelihood tree was performed in RAxML (Stamatakis 2006) using the standard search algorithm (data partitioned, GTRMIX model, 2000 bootstrap replications). Significance threshold was set above 0.95 for posterior probability (PP) and 70% bootstrap proportions (BP).

## Results

### Phylogeny

**Figure 1.**
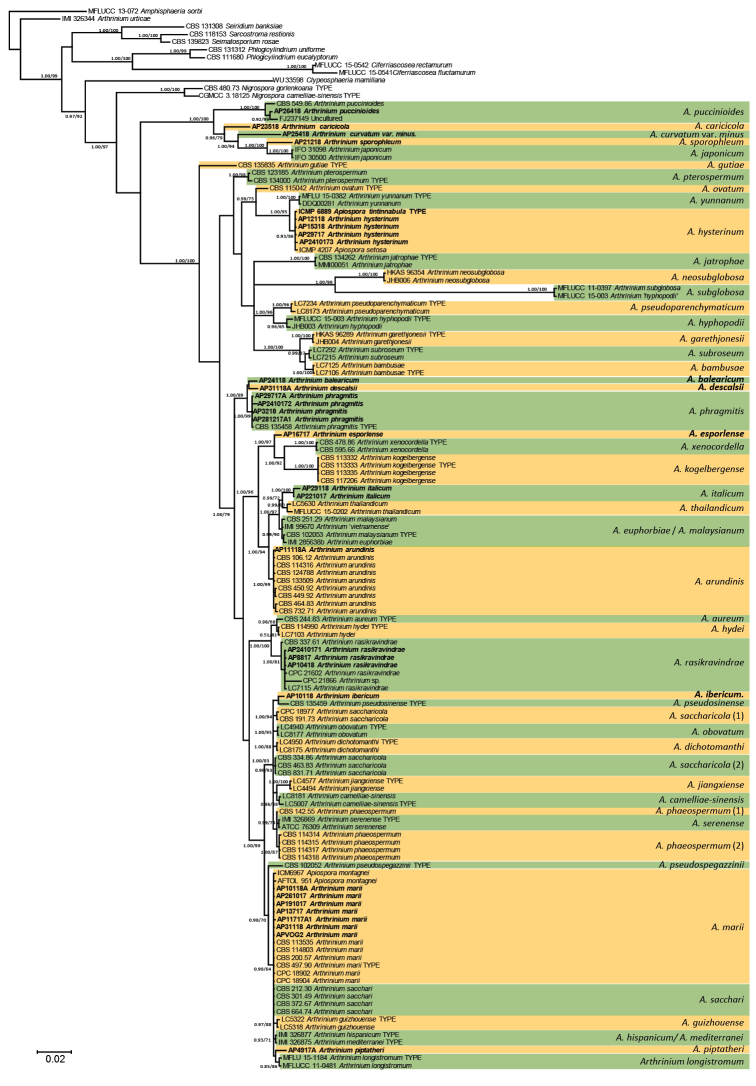
50% majority rule consensus phylogram obtained in MrBayes from 25725 trees after the analysis of ITS rDNA, 28S rDNA, tef1 and tub2 sequences (introns excluded) of the family Apiosporaceae. Nodes were annotated if supported by > 70% ML BP or > 0.95 bayesian PP, but non-significant support values are exceptionally represented inside parentheses. Bold names represent samples sequenced in the present study.

The analysis of ITS, 28S rDNA, tef1 and tub2 data from the entire family Apiosporaceae (Fig. 1) produced a phylogeny with two main significantly supported clades: 1) composed of *A.puccinioides*, *A.japonicum* and newly sequenced specimens matching the species *A.cariciola*, A.curvatumvar.minus, and *A.sporophleum*, and 2) a second clade containing all other sequences of *Arthrinium* and *Apiospora*. Among the other specimens analyzed, some matched the genetic concept of *A.hysterinum*, *A.phragmitis*, *A.arundinis*, *A.rasikravindrae*, or *A.marii*. Five new lineages were also found, which are formally proposed as new taxa below.

**Figure 2.**
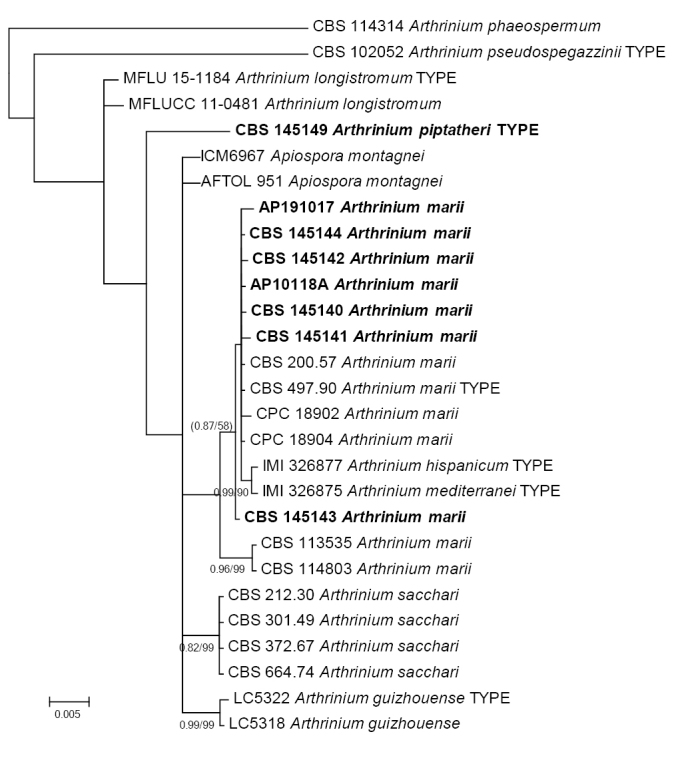
50% majority rule consensus phylogram obtained in MrBayes from 6750 trees after the analysis of ITS rDNA, 28S rDNA, tef1 and tub2 sequences (introns included) of the /sacchari clade. Nodes were annotated if supported by > 70% ML BP or > 0.95 bayesian PP, but non-significant support values are exceptionally represented inside parentheses. Bold names represent samples sequenced in the present study.

The analysis of ITS, 28S rDNA, tef1 and tub2 of the species around *A.sacchari* (/sacchari clade) (Fig. 2) showed that the clade of *A.marii* contains the types of *A.hispanicum* and *A.mediterranei*, but receives low overall support, maybe because of the incomplete data from these two species. Samples CBS 113535 and CBS 114803 were identified as *A.marii* too, but seem to represent an independent lineage.

**Table 1.** Details of strains included in this study. Types are in bold.

**Species**	**Isolate**	**CBS culture**	**Herbarium code**	**Host**	**ITS rDNA**	**28S rDNA**	**tef1**	**tub2**
* A. arundinis *	AP11118A	CBS 145128	MA-Fungi 91722	*Bambusa* sp.	MK014835	MK014868	MK017945	MK017974
***A.balearicum*, holotype**	**AP24118**	**CBS 145129**	**MA-Fungi 91723**	***Undetermined poaceae***	**MK014836**	**MK014869**	**MK017946**	**MK017975**
* A. caricicola *	AP23518	CBS 145127	MA-Fungi 91725	* Carex ericetorum *	MK014838	MK014871	MK017948	MK017977
A.curvatumvar.minus.	AP25418	CBS 145131	MA-Fungi 91726	hojas de *Carex* sp.	MK014839	MK014872	MK017949	MK017978
***A.descalsii*, holotype**	**AP31118A**	**CBS 145130**	**MA-Fungi 91724**	*** Ampelodesmos mauritanicus ***	**MK014837**	**MK014870**	**MK017947**	**MK017976**
***A.esporlense*, holotype**	**AP16717**	**CBS 145136**	**MA-Fungi 91727**	*** Phyllostachys aurea ***	**MK014845**	**MK014878**	**MK017954**	**MK017983**
* A. hysterinum *	AP15318	CBS 145132	MA-Fungi 91728	* Phyllostachys aurea *	MK014840	MK014873	MK017950	MK017979
			ICMP6889	* Bambusa *	MK014841	MK014874	MK017951	MK017980
	AP29717	CBS 145133	MA-Fungi 91729	* Phyllostachys aurea *	MK014842	MK014875	MK017952	MK017981
	AP2410173	CBS 145134	MA-Fungi 91730	* Phyllostachys aurea *	MK014843	MK014876		
	AP12118	CBS 145135	MA-Fungi 91731	* Phyllostachys aurea *	MK014844	MK014877	MK017953	MK017982
***A.ibericum*, holotype**	**AP10118**	**CBS 145137**	**MA-Fungi 91732**	*** Arundo donax ***	**MK014846**	**MK014879**	**MK017955**	**MK017984**
***A.italicum*, holotype**	**AP221017**	**CBS 145138**	**MA-Fungi 91733**	*** Arundo donax ***	**MK014847**	**MK014880**	**MK017956**	**MK017985**
	AP29118	CBS 145139	MA-Fungi 91734	* Phragmites australis *	MK014848	MK014881	MK017957	MK017986
* A. marii *	AP13717	CBS 145140	MA-Fungi 91735	* Arundo donax *	MK014849	MK014882	MK017958	MK017987
	AP10118A			* Phragmites australis *	MK014850	MK014883	MK017959	MK017988
	AP11717A	CBS 145141	MA-Fungi 91737	* Ampelodesmos mauritanicus *	MK014851	MK014884	MK017960	MK017989
	AP191017		MA-Fungi 91738	* Phragmites australis *	MK014852	MK014885	MK017961	MK017990
	AP261017	CBS 145142	MA-Fungi 91739	* Piptatheri miliaceum *	MK014853	MK014886	MK017962	MK017991
	Vog2	CBS 145143	MA-Fungi 91740	* Phragmites australis *	MK014854	MK014887	MK017963	MK017992
	AP31118	CBS 145144	MA-Fungi 91736	* Ampelodesmos mauritanicus *	MK014855	MK014888	MK017964	MK017993
* A. phragmitis *	AP281217A1	CBS 145145	MA-Fungi 91741	* Phragmites australis *	MK014856	MK014889	MK017965	MK017994
	AP2410172A	CBS 145146	MA-Fungi 91742	* Phragmites australis *	MK014857	MK014890	MK017966	MK017995
	AP3218	CBS 145147	MA-Fungi 91743	* Phragmites australis *	MK014858	MK014891	MK017967	MK017996
	AP29717A	CBS 145148	MA-Fungi 91744	* Arundo donax *	MK014859	MK014892	MK017968	MK017997
***A.piptatheri*, holotype**	**AP4817A**	**CBS 145149**	**MA-Fungi 91745**	*** Piptatherum miliaceum ***	**MK014860**	**MK014893**	**MK017969**	
* A. puccinioides *	AP26418	CBS 145150	MA-Fungi 91746	* Carex arenaria *	MK014861	MK014894	MK017970	MK017998
* A. rasikravindrii *	AP8817	CBS 145151	MA-Fungi 91747	* Phyllostachys aurea *	MK014862	MK014895		
	AP10418	CBS 145152	MA-Fungi 91748	* Phyllostachys aurea *	MK014863	MK014896	MK017971	MK017999
	AP2410171	CBS 145153		* Phyllostachys aurea *	MK014864	MK014897	MK017972	MK018000
* A. sporophleum *	AP21118	CBS 145154	MA-Fungi 91749	*Juncus* sp.	MK014865	MK014898	MK017973	MK018001

**Table 2.** Details of all strains included in the phylogenetic analyses. Sequences generated in this study are shown in bold.

**Species**	**voucher/culture**	**ITS rDNA**	**28S rDNA**	**tub2**	**tef1**
* Apiospora setosa *	ICMP 4207		DQ368631	DQ368620	
*** Apiospora tintinnabula ***	**ICMP6889**	**MK014841**	**MK014874**	**MK017951**	**MK01980**
*Arthrinium ‘vietnamense*’	IMI 99670	KX986096	KX986111	KY019466	
* Arthrinium arundinis *	CBS 106 12	KF144883	KF144927	KF144973	KF145015
* Arthrinium arundinis *	**CBS 145128**	**MK014835**	**MK014868**	**MK017945**	**MK017974**
* Arthrinium arundinis *	CBS 449 92	KF144887	KF144931	KF144977	KF145019
* Arthrinium arundinis *	CBS 450 92	AB220259	KF144932	KF144978	KF145020
* Arthrinium arundinis *	CBS 124788	KF144885	KF144929	KF144975	KF145017
* Arthrinium arundinis *	CBS 133509	KF144886	KF144930	KF144976	KF145018
* Arthrinium arundinis *	CBS 114316	KF144884	KF144928	KF144974	KF145016
* Arthrinium arundinis *	CBS 464 83	KF144888	KF144933	KF144979	KF145021
* Arthrinium arundinis *	CBS 732 71	KF144889	KF144934	KF144980	KF145022
* Arthrinium arureum *	CBS 24483	AB220251	KF144935	KF144981	KF145023
*** Arthrinium balearicum ***	**CBS 145129**	**MK014836**	**MK014869**	**MK017946**	**MK017975**
* Arthrinium camelliae-sinensis *	LC8181	KY494761	KY494837	KY705229	KY705157
* Arthrinium camelliae-sinensis *	LC5007	KY494704	KY494780	KY705173	KY705103
* Arthrinium caricicola *	**CBS 145127**	**MK014838**	**MK014871**	**MK017948**	**MK017977**
Arthrinium curvatum var. minus	**CBS 145131**	**MK014839**	**MK014872**	**MK017949**	**MK017978**
*** Arthrinium descalsii ***	**CBS 145130**	**MK014837**	**MK014870**	**MK017947**	**MK017976**
* Arthrinium dichotomanthi *	LC8175	KY494755	KY494831	kY705223	KY705151
* Arthrinium dichotomanthi *	LC4950	KY494697	KY494773	KY705167	KY705096
*** Arthrinium esporlense ***	**CBS 145136**	**MK014845**	**MK014878**	**MK017954**	**MK017983**
* Arthrinium euphorbiae *	IMI 285638b	AB220241	AB220335	AB220288	
* Arthrinium garethjonesii *	JHB004	KY356096	KY356091		
* Arthrinium garethjonesii *	HKAS 96289	NR_154736	NG_057131		
* Arthrinium guizhouense *	LC5322	KY494709	KY494785	KY705178	KY705108
* Arthrinium guizhouense *	LC5318	KY494708	KY494784	KY705177	KY705107
* Arthrinium hydei *	CBS 114990	KF144890	KF144936	KF144982	KF145024
* Arthrinium hydei *	LC7103	KY494715	KY4947911	KY705183	KY705114
* Arthrinium hyphopodii *	MFLUCC 15-003	NR_154699			
* Arthrinium hyphopodii *	JHB003 Art	KY356098	KY356093		
*** Arthrinium hysterinum ***	**CBS 145133**	**MK014842**	**MK014875**	**MK017952**	**MK01981**
*** Arthrinium hysterinum ***	**CBS 145135**	**MK014844**	**MK014877**	**MK017953**	**MK01982**
*** Arthrinium hysterinum ***	**CBS 145132**	**MK014840**	**MK014873**	**MK017950**	**MK01879**
*** Arthrinium hysterinum ***	**CBS 145134**	**MK015843**	**MK014876**		
*** Arthrinium ibericum ***	**CBS 145137**	**MK014846**	**MK014879**	**MK017955**	**MK017984**
*** Arthrinium italicum ***	**CBS 145138**	**MK014847**	**MK014880**	**MK017956**	**MK017985**
*** Arthrinium italicum ***	**CBS 145139**	**MK014848**	**MK014881**	**MK017957**	**MK017986**
* Arthrinium japonicum *	IFO30500	AB220262	AB220309	AB220309	
* Arthrinium japonicum *	IFO31098	AB220264	AB220311	AB220311	
* Arthrinium jatrophae *	CBS 134262	NR_154675			
* Arthrinium jatrophae *	MMI00051	AB743995			
* Arthrinium jiangxiense *	LC4577	KY494693	KY494769	KY705163	KY705092
* Arthrinium jiangxiense *	LC4494	KY494691	KY494766	KY705160	KY705089
* Arthrinium kogelbergense *	CBS 113332	KF144891	KF144937	KF144983	KF145025
* Arthrinium kogelbergense *	CBS 113333	KF144892	KF144938	KF144984	KF145026
* Arthrinium kogelbergense *	CBS 113335	KF144893	KF144939	KF144985	KF145027
* Arthrinium kogelbergense *	CBS 117206	KF144895	KF144941	KF144987	KF145029
* Arthrinium longistromum *	MFLU 15-1184	NR_154716			
* Arthrinium longistromum *	MFLUCC 11-0481	KU940141	KU863129		
* Arthrinium malaysianum *	CBS 102053	KF144896	KF144942	KF144988	KF145030
* Arthrinium malaysianum *	CBS 251.29	KF144897	KF144943	KF144989	KF145031
* Arthrinium marii *	CPC 18902	KF144901	KF144948		
* Arthrinium marii *	**CBS 145140**	**MK014849**	**MK014882**	**MK017958**	**MK017987**
* Arthrinium marii *	CBS 114803	KF144899	KF144945	KF144991	KF145033
* Arthrinium marii *	CBS 113535	KF144898	KF144944	KF144990	KF145032
* Arthrinium marii *	**CBS 145141**	**MK014851**	**MK014884**	**MK017960**	**MK017989**
* Arthrinium marii *	**CBS 145142**	**MK014853**	**MK014886**	**MK017962**	**MK017991**
* Arthrinium marii *	**CBS 145143**	**MK014854**	**MK014887**	**MK017963**	**MK017992**
* Arthrinium marii *	**CBS 145144**	**MK014855**	**MK014888**	**MK017964**	**MK017993**
* Arthrinium mediterranei *	IMI 326875	AB220243	AB220337	AB220290	
* Arthrinium neosubglobosa *	HKAS 96354	NR_154737	NG_057131		
* Arthrinium neosubglobosa *	JHB006	KY356089	KY356094		
* Arthrinium obovatum *	LC8177	KY494757	KY494833	KY705225	KY705153
* Arthrinium obovatum *	LC4940	KY494696	KY494772	KY705166	KY705095
* Arthrinium ovatum *	CBS 115042	KF144903	KF144950	KF144995	KF145037
* Arthrinium phaeospermum *	CBS 114317	KF144906	KF144953	KF144998	KF145040
* Arthrinium phaeospermum *	CBS 114318	KF144907	KF144954	KF144999	KF145041
* Arthrinium phaeospermum *	CBS 114315	KF144905	KF144952	KF144997	KF145039
* Arthrinium phaeospermum *	CBS 114314	KF144904	KF144951	KF144996	KF145038
* Arthrinium phragmitis *	**CBS 145145**	**MK014856**	**MK014889**	**MK017965**	**MK017994**
* Arthrinium phragmitis *	**CBS 145146**	**MK014857**	**MK014890**	**MK017966**	**MK017995**
* Arthrinium phragmitis *	CBS 135458	KF144909	KF144956	KF145001	KF145043
* Arthrinium phragmitis *	**CBS 145147**	**MK014858**	**MK014891**	**MK017967**	**MK017996**
* Arthrinium phragmitis *	**CBS 145148**	**MK014859**	**MK014892**	**MK017968**	**MK017997**
*** Arthrinium piptatheri ***	**CBS 145149**	**MK014860**	**MK014893**	**MK017969**	
* Arthrinium pseudosinense *	CBS 135459	KF144910	KF144957		KF145044
* Arthrinium pseudospegazzinii *	CBS 102052	KF144911	KF144958	KF145002	KF145045
* Arthrinium pterospermum *	CBS 123185	KF144912	KF144959	KF145003	
* Arthrinium pterospermum *	CBS 134000	KF144913	KF144960	KF145004	KF145046
* Arthrinium puccinioides *	**CBS 145150**	**MK014861**	**MK014894**	**MK017970**	**MK017998**
* Arthrinium rasikravindrae *	CBS 33761	KF144914	KF144961		
* Arthrinium rasikravindrae *	**CBS 145151**	**MK014862**	**MK014895**		
* Arthrinium rasikravindrae *	CPC 21602	KF144915			
* Arthrinium rasikravindrae *	**CBS 145152**	**MK014863**	**MK014896**	**MK017971**	**MK017999**
* Arthrinium rasikravindrae *	LC7115	KY494721	KY494797	KY708159	KY705118
* Arthrinium rasikravindrae *	**CBS 145153**	**MK014864**	**MK014897**	**MK017972**	**MK018000**
* Arthrinium sacchari *	CBS 30149	KF144917	KF144963	KF145006	KF145048
* Arthrinium sacchari *	CBS 21230	KF144916	KF144962	KF145005	KF145047
* Arthrinium sacchari *	CBS 66474	KF144919	KF144965	KF145008	KF145050
* Arthrinium sacchari *	CBS 37267	KF144918	KF144964	KF145007	KF145049
*Arthriniumsaccharicola* (1)	CBS 19173	KF144920	KF144966	KF145009	KF145051
*Arthriniumsaccharicola* (1)	CPC 18977	KF144923			
*Arthriniumsaccharicola* (2)	CBS 33486	AB220257	KF144967	KF145010	KF145052
*Arthriniumsaccharicola* (2)	CBS 83171	KF144922	KF144969	KF145012	KF145054
*Arthriniumsaccharicola* (2)	CBS 46383	KF144921	KF144968	KF145011	KF145053
* Arthrinium serenense *	ATCC 76309	AB220240	AB220334	AB220287	
* Arthrinium serenense *	IMI 326869	AB220250	AB220344	AB220297	
*** Arthrinium sporophleum ***	**CBS 145154**	**MK014865**	**MK014898**	**MK017973**	**MK018001**
* Arthrinium subglobosa *	MFLUCC 11-0397	KR069112	NG_057070		
*Arthriniumsubglobosa* (‘*hyphopodii*’)	MFLUCC 15-003	KR069111			
* Arthrinium subroseum *	LC7292	KY494752	KY494828	KY705220	KY705148
* Arthrinium subroseum *	LC7215	KY494740	KY494816	KY705208	KY705236
* Arthrinium thailandicum *	LC5630	KY494714	KY494790	KY806200	KY705113
* Arthrinium thailandicum *	MFLUCC 15-0202	KU940145	KU863133		

### Taxonomy

#### Arthrinium balearicum

Taxon classificationFungiXylarialesApiosporaceae

Pintos & P. Alvarado sp. nov.

828866

Fig. 3

##### Etymology.

Refers to the Balearic Islands (Spain), where the holotype was found.

##### Diagnosis.

Sexual morph: *Stromata* forming black, linear, confluent raised areas on host surface, with the longer axis broken at the apex, (500–)600–1500(–2000) µm × (200–)320–450(–500) µm (*n* = 20). *Ascomata* globose to subglobose, with flattened base, blackish brown, (120–) 140–180 (–200) µm in diameter (*n* = 30). *Peridium* 8–15 µm thick, consisting of 4–5 layers of cells arranged in *textura angularis*, externally dark brown, hyaline in the inner part. *Ostiole* single, central, 30–60 µm in diameter, with a periphysate channel 20–30 µm long. *Peryphises* broad, colourless. *Hamathecium* composed of dense hypha-like, broad septate paraphyses, deliquescing early, 4–6 µm thick. *Asci* 8-spored, unitunicate, clavate, broadly cylindrical, with an inconspicuous pedicel, rounded apex, thin-walled, without an apical apparatus, measuring (77–)80–98(–105) × (14–)15–19(–21) µm (*n* = 22). *Ascospores* 1–3-seriate, hyaline, apiospore smooth-walled, fusiform, elliptical, reniform, straight or curved, bicellular, wider at the center of the longest cell, measuring (23–)26–30(–32) × (7–)9–10(–12) µm (*n* = 35), basal cell 3–6 µm long, sometimes containing a droplet. Asexual morph: not observed. Culture characteristics: colonies flat spreading on MEA 2%, with moderate aerial mycelium, reverse withish.

##### Type.

Spain: Balearic Islands: Mallorca, Llucmajor, on undetermined Poaceae, 24 Jan. 2018, *A. Pintos* (MA-Fungi 91723 holotype, AP24118 isotype, CBS 145129 ex-type culture).

##### Notes.

*Arthriniumbalearicum* is related with *A.descalsii*, but has some genetic differences with this species having only 93% (482/518 bp) of its ITS rDNA, 99% (821/823 bp) of 28S rDNA, 97% (688/707 bp) of tef1, and 98% (406/413 bp) of tub2 similar. It is also phylogenetically close to *A.phragmitis*, a species with a similar ascospore size, (23–)26–30(–32) × (7–)9–10(–12) µm in *A.balearicum* and (22–)23–28(–30) µm × (6–)7–9(–10) µm in *A.phragmitis*. Unfortunately, the asexual morph of *A.balearicum* could not be studied to compare it with that of *A.phragmitis*.

**Figure 3.**
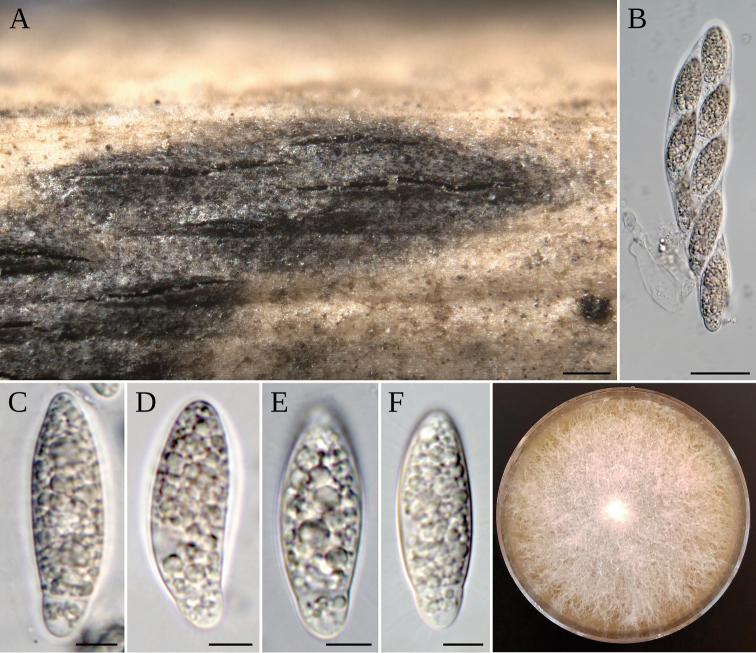
*A.balearicum***A** stromata on host; **B** asci **C–F** ascospores **G** colony on MEA. Scale bars: 200 µm (**A**); 20 µm (**B**); 5 µm (**C–F**).

#### Arthrinium caricicola

Taxon classificationFungiXylarialesApiosporaceae

Kunze & J.C. Schmidt, Mykologische Hefte (Leipzig) 1: 9 (1817)

Fig. 4

##### Description.

Asexual morph: *colonies* on the host punctiform, pulvinate, 140–400 µm in diameter, blackish brown. *Mycelium* formed by hyaline smooth, branched hyphae, 2–5 µm in diameter. *Conidiophore mother cells* arising from a superficial or erumpent mycelial mat, subspherical to lageniform in shape, hyaline with brown pigments at the base, measuring (4–)5–7(–8) × (8–)9–11(–12) µm (*n* = 45). *Conidiophores* erect or ascending, simple, straight or flexuous, cylindrical, smooth-walled, colourless excepting for the thick, brown to dark brown, transversal septa, 15–100 × 3–5 µm (*n* = 50). *Conidia* fusiform or broadly spindle-shaped, smooth-walled, broader at the middle, tapering towards the narrowly rounded ends, dark brown with a hyaline rim, (37–)44–51(–55) µm in frontal view, (8–)9–11(–12) µm in side view (*n* = 50). *Sterile cells* smaller, 15–19 × 10–13 µm, and paler than conidia, bicuspidate or irregularly lobed. *Culture characteristics*: flat colonies spreading on MEA 2%, with moderately abundant, white cottony aerial mycelium, reverse whitish too, circular in shape with irregular edge.

##### Notes.

The conidia of *A.caricicola* and *A.japonicum* have a similar fusiform shape and length, but differ in width ((8–)9–11(–12) µm vs 12–16(–20) µm). Conidia of *A.mytilimorphum* have also a similar shape, but turns out shorter and thinner (20–30 × 6–8.5 µm). The morphological characters of the syntype of *A.caricicola* deposited by Fries in the Herbarium of Uppsala University as Fung. Scleromyc. Suecici, fully match the specimen collected in this study. The closely related species *A.sporophleum* has very different lemon-shaped conidia, while those of A.curvatumvar.minus are curved, and those of *A.puccinioides* are polygonal.

##### Specimens examined.

Germany: *Brandenburg*: south of Liberose, on dead leaves of *Carexericetorum*, 14 May 2018, *R. Jarling* (MA-Fungi 91725).

**Figure 4.**
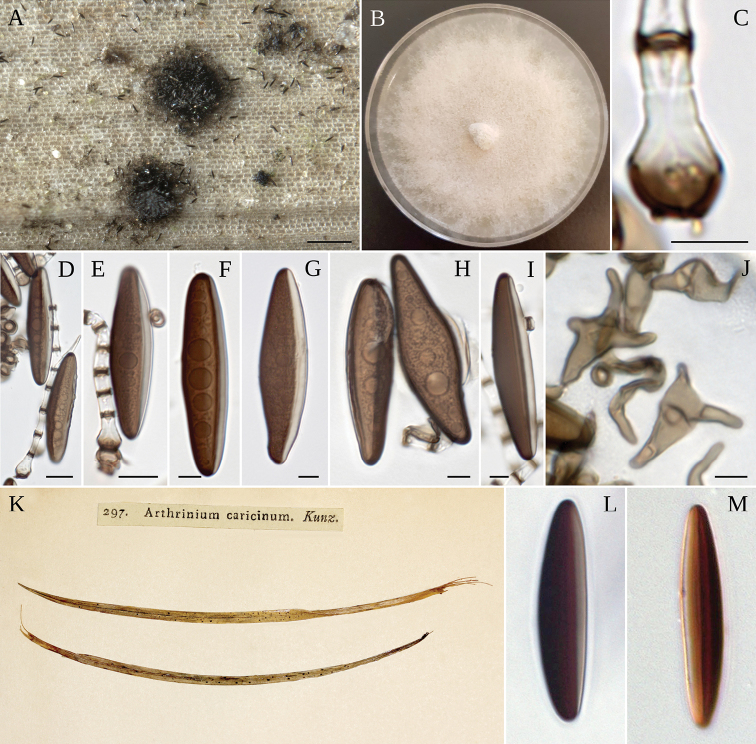
*A.caricicola***A** colony on host **B** colony on MEA**C** conidiophore mother cell **D, E** conidiophore mother cell, conidiophore bearing conidia, conidia **F–H** conidia **I** conidia with scar **J** lobate sterile cells. Scale bars: 200 µm (**A**); 5 µm (**C–I**); 10 µm (**J**). **K***A.caricicola* syntype, colonies on host; **L, M** conidia.

#### Arthrinium curvatum var. minus

Taxon classificationFungiXylarialesApiosporaceae

M.B. Ellis, Trans. Brit. Mycol. Soc. 34: 501 (1951)

Fig. 5

Physalospora scirpi Arx, Gen. Fungi Sporul. Cult. (Lehr): 116 (1970).
Pseudoguignardia scirpi Gutner, Mater. Mikol. Fitopat. Ross. 6(1): 311 (1927).

##### Description.

Asexual morph: *Colonies* are compact, round, dark to black, 80–320 in diameter. *Mycelium* is composed of hyaline to pale brown smooth hyphae 2–7 µm in diameter. *Conidiophore mother cells* spherical to lageniform, hyaline with brown pigments at the base, measuring (4–)5–7(–8) × (4–)5–6(–7) µm (*n* = 30). *Conidiophores* cylindrical unbranched, straight or flexuous, hyaline and smooth walled, with a single brown transversal septa, measuring 30–100 × 2–4 µm. (*n* = 30). *Conidiogenous cells* cylindrical 1–1.5 × 1–1.5 µm (*n* = 20). *Conidia* borne along the sides of conidiophores, curved, rounded at the ends, brown, with a hyaline germ slit and a clearly visible scar, (8–)9–10(–11) µm long in frontal view, (5–)6–7(–8) µm in side view (*n* = 30). *Sterile cells* rounded, paler than conidia. *Culture characteristics* flat colonies spreading on MEA 2% with moderate aerial mycelium, reverse withish.

##### Notes.

Arthriniumcurvatumvar.minus can be confused with A.curvatumvar.curvatum , but conidia of var. minus measure (8–)9–10(–11) × (5–)6–7(–8) µm, while those of A.curvatumvar.curvatum measure 11–15 × 6–8 µm. Gutner (1927) described *Pseudoguignardiascirpi*, a sexual morph of *A.curvatum*, later combined as *Physalosporascirpi* (Arx 1970). Arthriniumcurvatumvar.minus is closely related with *A.sporophleum* (with lemon-shaped conidia) and *A.japonicum* (with larger fusiform conidia) and to a lesser extent also with *A.caricicola* (with larger fusiform conidia) and *A.puccinioides* (with polygonal conidia). Ellis et al. (1951) described A.curvatumvar.minus, a taxon with similarly shaped but smaller conidia than *A.curvatum*. The specimen studied in the present work matches the shape and size of conidia reported by Ellis et al. (1951) for A.curvatumvar.minus, rather than those of A.curvatumvar.curvatum.

##### Specimens examined.

Germany: Brandenburg: south of Liberose, on dead leaves of *Carex* sp., 28 Mar. 2018, *R. Jarling* (MA-Fungi 91726).

**Figure 5.**
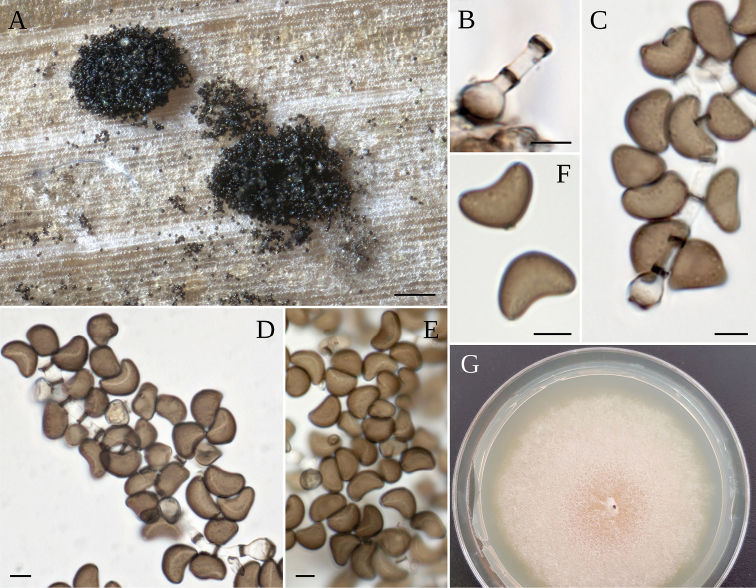
A.curvatumvar.minus**A** colony on host **B** conidiophore mother cell **C, D** conidiophore mother cell, conidiophore bearing conidia **E, F** curved conidia **G** colony on MEA. Scale bars: 200 µm (**A**); 5 µm (B–F).

#### Arthrinium descalsii

Taxon classificationFungiXylarialesApiosporaceae

Pintos & P. Alvarado sp. nov.

828867

Fig. 6

##### Etymology.

Named to honor the eminent mycologist Enric Descals Callisen.

##### Diagnosis.

Sexual morph: *Stromata* forming black fusiform spots that merge with each other with age, forming an erumpent black mass visible at the naked eye, 2–10 × 0.2–0.5 mm in size, with the long axis broken at the top revealing the ostioles of pseudothecia. *Ascomata* pseudothecia, subglobose with a flattened base, arranged in rows, brown to dark brown, 150–220 μm high × 150–250 μm wide (*n* = 20). *Peridium* with several layers of cells arranged in *textura angularis*, with a conspicuous ostiole 50–80 μm in diameter, periphysate. *Hamathecium* paraphyses hyphae-like, septate, hyaline. *Asci* cylindrical, clavate, with a short or indistinct pedicel, with rounded apices, measuring (73–)82–95(–111) × (16–)17–20(–23) μm (*n* = 30). *Ascospores* uniseriate to biseriate, hyaline, smooth-walled, apiosporic, composed of a large curved upper cell and smaller lower cell, fusiform to slightly curved in shape with narrowly rounded ends, guttulated, sometimes with a thick gelatinous sheath, (17–)18–22(–24) × (6–)7–9(–10) μm, and a basal cell 3–5 μm (*n* = 45). Asexual morph: *Mycelium* hyaline, septate, branched, hyphae 1.5–4.5 μm in diameter *Conidiophores* reduced to the conidiogenous cells. *Conidiogenous cells* solitary on hyphae, ampuliform, hyaline to brown, 5 × 4 μm. *Conidia* brown, smooth, guttulate, globose to ellipsoid (5–)7(–8) µm long (*n* = 20) in face view, lenticular with a paler equatorial slit and 6-7 μm long in side view (*n* = 10). *Sterile cells* elongated, sometimes mixed among conidia. *Culture characteristics*: ascospores germinating on MEA 2% within 24–48 h. *Colonies* flat, spreading, with sparse aerial mycelium, pale siena.

##### Notes.

*Arthriniumdescalsii* is closely related with *A.phragmitis* and *A.balearicum*. It was found in the Mediterranean grass *Ampelodesmosmauritanicus*, although additional samples are needed before concluding if it could be exclusively associated with this endemic host. Ascospore size is often smaller than that of *A.balearicum*, (23–)26–30(–32) × (7–)9–10(–12) µm, but it matches that reported in the protologue of *A.phragmitis*, (20–)22–24(–25) × (7–)8–9(–10) µm. However, the conidiophores of *A.descalsii* are reduced to conidiogenous cells, while those of *A.phragmitis* measure about 10–45 × 1.5–2 µm, and conidia are slightly smaller in face view, measuring (5–)7(–8) µm long in *A.descalsii* and up to 8–10(–11) µm in *A.phragmitis*.

##### Type.

Spain: Balearic Islands: Mallorca, es Capdella, on dead stems of *Ampelodesmosmauritanicus*, 31 Jan. 2018, *A. Pintos* (MA-Fungi 91724 holotype, AP31118A isotype, CBS 145130 ex-type culture).

**Figure 6.**
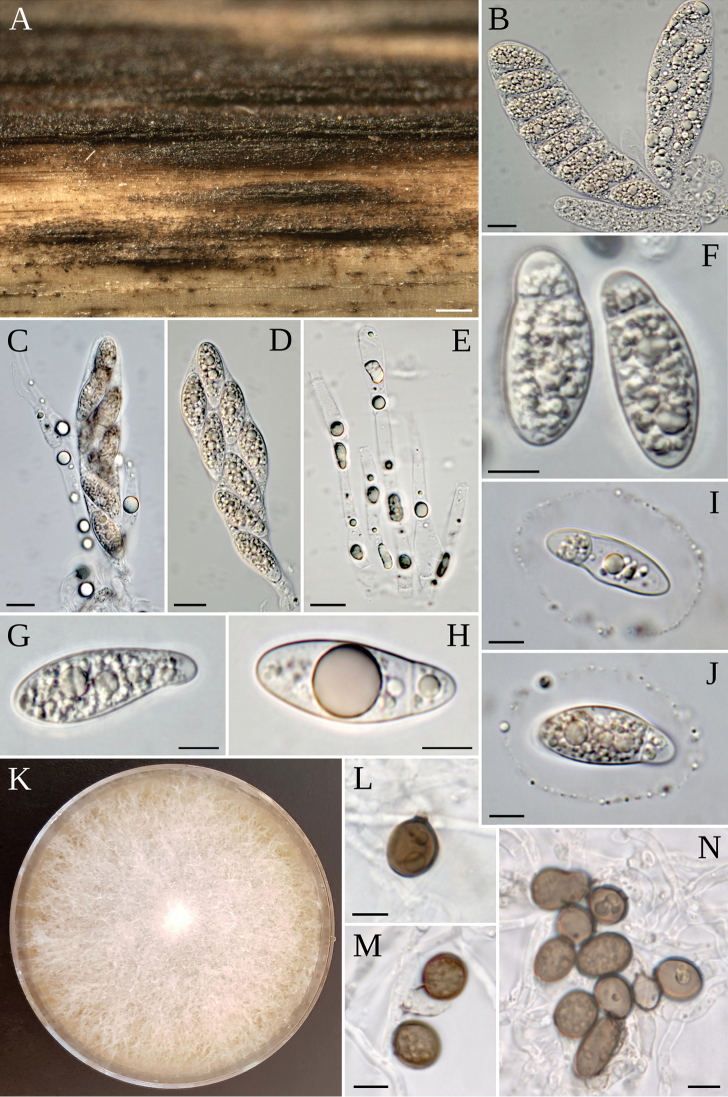
*A.descalsii***A** stromata on host **B–D** asci with ascospores **E** paraphyses **F, G** ascospores **I, J** ascospores with sheath **K** colony on MEA 2%; coniogenous cell giving rise to conidia; conidiogenous cells giving rise to conidia and conidia cluster **G** conidia. Scale bars: 200 µm (**A**); 10 µm (**B–E**); 5 µm (**F–J**); 5 µm (**L–N**).

#### Arthirnium esporlense

Taxon classificationFungiXylarialesApiosporaceae

Pintos & P. Alvarado sp. nov.

828868

Fig. 7

##### Etymology.

In reference to Esporles, the village of Mallorca (Spain) where it was found.

##### Diagnosis.

Asexual morph: *Mycelium* consisting of smooth, hyaline, branched septate hyphae about 1.5–4 µm in diameter. *Conidiophores* reduced to conidiogeous cells. *Conidiogenous cells* polyblastic, aggregated in clusters on hyphae, smooth, hyaline to pale brown, ampuliform, cylindrical or lageniform, measuring 4–22 × 4–8 μm. *Conidia* brown, smooth, globose with a pale equatorial slit and (8–)9–12(–13) µm long in frontal view, lenticular and 6–8 μm long in side view (*n* = 30). *Sterile cells* elongated, sometimes mixed among conidia, paler than them. *Culture characteristics*: colonies flat, spreading, with moderate aerial mycelium, on MEA 2% surface white with yellowish patches, reverse concolour with age.

##### Type.

Spain: Balearic Islands: Mallorca, Esporles, on dead culms of *Phyllostachysaurea*, 16 July 2017, *A. Pintos* (MA-Fungi 91727 holotype, AP16717 isotype, CBS 145136 ex-type culture).

##### Notes.

*Arthriniumesporlense* is closely related with *A.xenocordella* and *A.kogelbergense*. However, *A.esporlense* does not produce brown setae as *A.xenocordella*, a species until now known only from soil samples (Crous and Groenewald 2013). *Arthriniumesporlense* morphologically differs from *A.kogelbergense* by producing slightly bigger conidiogenous cells (4–22 × 4–8 μm vs 5–12 × 4–5 μm). These three species are genetically related (1.00 PP, 96 BP) to the group formed by *A.arundinis*, *A.thailandicum* D.Q. Dai & K.D. Hyde, *A.malaysianum* and the new species *A.italicum* proposed below.

**Figure 7.**
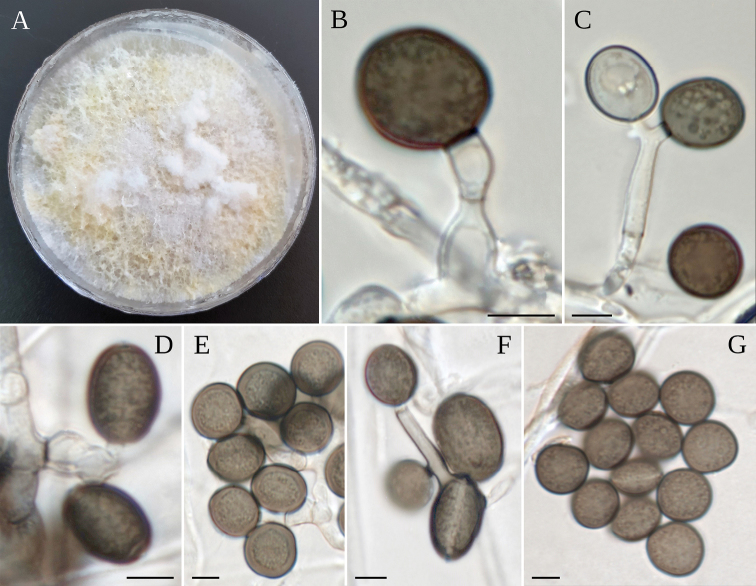
*A.esporlense***A** colony on MEA**B–F** coniogenous cell giving rise to conidia **G** conidia. Scale bars: 5 µm (**B–G**).

#### Arthrinium hysterinum

Taxon classificationFungiXylarialesApiosporaceae

(Sacc.) P.M. Kirk, Trans. Brit. Mycol. Soc. 86: 409 (1986)

Fig. 8

Melanconium hysterinum Sacc., Bolm Soc. broteriana, Coimbra, sér. 1 11: 21 (1893) [Basionym].
Scyphospora hysterina (Sacc.) Sivan., Trans. Brit. Mycol. Soc. 81: 331 (1983).
Melanconium bambusae Turconi, Atti Ist. bot. R. Univ. Pavia, sér. 2 16: 251 (1916).
Scirrhia bambusae Turconi, Atti Ist. bot. R. Univ. Pavia, sér. 2 16: 531 (1916).
Scirrhodothis bambusae (Turconi) Trotter, in Saccardo, Syll. Fung. 24: 611 (1926).
Placostroma bambusae (Turconi) R. Sprague, Diseases Cereals Grasses N. Amer.: 121 (1950).
Apiospora bambusae (Turconi) Sivan., Trans. Brit. Mycol. Soc. 81: 331 (1983).
Scyphospora phyllostachydis L.A. Kantsch., Bolêz. Rast. 17: 88 (1928).
Cordella johnstonii M.B. Ellis, Mycol. Pap. 103: 31 (1965).
Apiospora setosa Samuels et al., New Zealand J. Bot. 19: 142 (1981).
Apiospora tintinnabula Samuels et al., New Zealand J. Bot. 19: 142 (1981).

##### Description.

Sexual morph: *Stromata* black, fusiform, forming rows of densely arranged perithecial ascomata parallel to the main axis of the host, measuring (400–) 600–2500(–3000) × (250–)320–450(–550) µm (*n* = 30). *Ascomata* globose to subglobose, with a flattened base, blackish brown, (130–)250–290(–320) µm in diameter (*n* = 30). *Peridium* consisting of 3 or 4 layers of cells arranged in *textura angularis*, dark brown in the external side, hyaline in the inside, ostiole single, central, 10–30 µm in diameter, with a periphysate channel 20–35 µm long. *Peryphises* broad, colourless. *Hamathecium* composed of dense hypha-like, broad septate paraphyses, early deliquescing. *Asci* 8-spored, unitunicate, clavate, broadly cylindrical, pedicel indistinct, apical rounded, thin-walled, without an apical apparatus, measuring (76–) 85–98(–115) × (20–)22–26(–28) µm (*n* = 22). *Ascospores* uni- to tri-seriate, hyaline, apiosporic, smooth-walled, fusiform, elliptical, reniform, straight or curved, smooth-walled, sometimes with an internal droplet, bicellular, the widest part located in the central part of the longest cell, some ascospores have a mucose sheath covering them, (28–)32–34(–38) × (8–)9–11(–13) (*n* = 35) µm, basal cell 5–7 µm. Asexual morph: *Mycelium* branched, septate. *Conidiomata* on host surrounding the stromata of the sexual phase, parallel to the longitudinal axis of the stem, subepidermal, opening by longitudinal splitting of the epidermis and revealing a black conidial mass, (450–) 630–950(–1000) × (275–)345–550 (–600) µm (*n* = 35). *Conidiophore mother cell* arising from the stroma, ampuliform, lageniform, cupulate or cylindrical, sometimes with granular pigments at the apex, (5)6–10(–16) × (3–)5–7(–8) µm (*n* = 24). *Conidiophores* basauxic, polyblastic, cylindrical, hyaline to light brown, smooth or with granular pigments in all their length, straight or flexuous, septate or not, sometimes exceeding 90 μm in length × 2–4 μm wide (*n* = 43). *Conidia* globose to obovoid, dark brown, with a central scar at the base, (15–)16–20(–21) in frontal view, (14–)15–18(–19) in side view (*n* = 40). *Sterile cells* gray, irregularly angled and lobed, (15–)17–41(–42) × (10–)14–23(–25) µm (*n* = 30). *Culture characteristics*: colonies in MEA 2% flat, spreading, first white and cottony, later became dark pink, *mycelium* branched, septate, hyaline, reverse dark.

##### Notes.

After the works of Samuels (1981), Sivanesan (1983), Kirk (1986) and Réblová et al. (2016), *Ap.bambusae*, *Ap.setosa* and *Ap.tintinnabula*, as well as *Scyphosporaphyllostachydis*, are all considered synonyms of *A.hysterinum*. *Arthriniumhysterinum* is phyllogenetically close to *A.yunnanum* D.Q. Dai & K.D. Hyde, but morphologically differs from the latter because of its thinner asci (76–115 × 20–28 vs 85–100 × 30–35 μm). In addition, *A.hysterinum* has longer conidiophores up to 90 μm long, and lobed sterile cells while in *A.yunnanum* conidiophores do not exceed 50 μm, and sterile cells are lacking.

##### Specimens examined.

New Zealand: Waikato: Paeroa, on dead culm of *Bambusa* sp., 28 Feb. 1980, *E.H.C. McKenzie & P.R. Johnston* (ICMP 6889 ex-type culture).

Spain: Galicia: Santiago de Compostela, on dead culms of *Phyllostachysaurea*, 12 Jan. 2018, *A. Pintos* (MA-Fungi 91731, AP12118). Balearic Islands: Mallorca, Esporlas, on dead culms of *Phyllostachysaurea*, 29 July 2017, *A. Pintos* (MA-Fungi 91729, AP29717). Mallorca, Jardin Botanico de Soller, on dead culms of *Phyllostachysaurea*, 24 Oct. 2017, *A. Pintos* (MA-Fungi 91730, AP2410173). Mallorca, Soller, on dead culms of *Phyllostachysaurea*, 15 Mar. 2018, *A. Pintos* (MA-Fungi 91728, AP15318).

**Figure 8.**
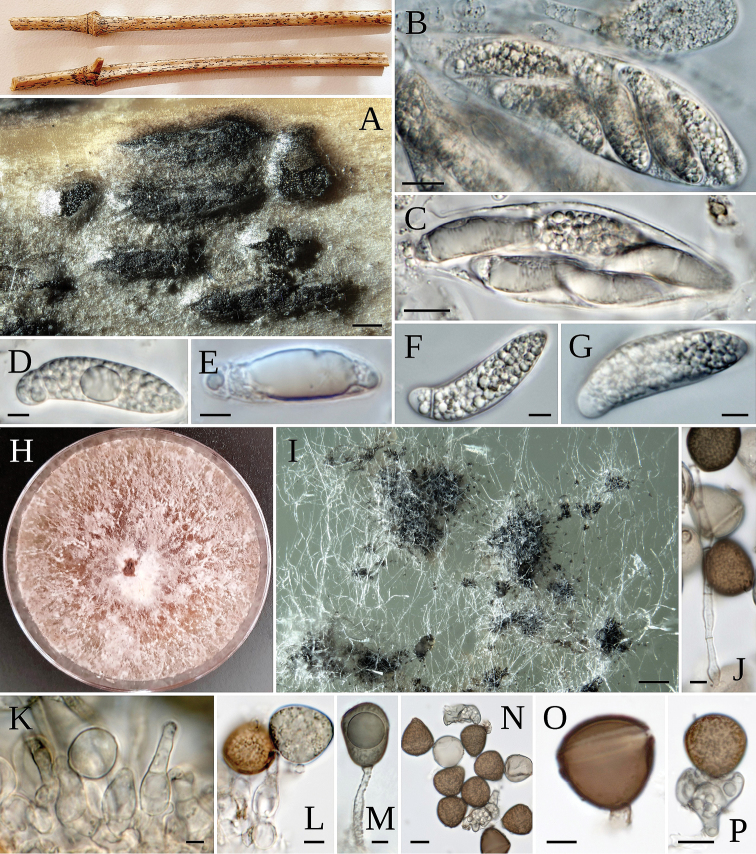
*A.hysterinum* lenticular-shaped colonies on host **A** stromata and conidiomata **B, C** asci **D–G** ascospores **H** colony on MEA**I** black masses of conidia in culture **K, L** conidiophore mother cell **M** rugose conidiogenous cell **N–P** conidia with lobate sterile cells **O** conidia. Scale bars: 200 µm (**A**); 10 µm (**B, C**); 5 µm (**D–G**); 200 µm (**I**); 5 µm (**K, M, O**); 10 µm (**P**).

#### Arthrinium ibericum

Taxon classificationFungiXylarialesApiosporaceae

Pintos & P. Alvarado sp. nov.

828869

Fig. 9

##### Etymology.

In reference to the Iberian Peninsula, where the holotype was collected.

##### Diagnosis.

Sexual morph: *Stromata* solitary to gregarious, immersed or semi-immersed, fusiform to ellipsoid in shape, black, with the long axis broken at the top, 2–5 × 0.5–1 mm. *Ascomata* perithecial, subglobose with a flattened base, arranged in rows, brown to dark brown, exudating a white cirrhus of ascospores, 170–300 µm in diameter and 200–300 µm high. *Peridium* consisting in 3 or 4 layers of cells arranged in *textura angularis*. *Ostiole* single, central, 12–30 µm in diameter, with a periphysate channel. *Hamathecium* composed of dense, septate, branched paraphyses. *Asci* 8-spored, clavate or cylindrical, lacking an apical apparatus, shortly pedicelate, measuring (82–)90–125(–128) × (14–)15–19(–21) μm (*n* = 30). *Ascospores* uniseriate to biseriate, hyaline, smooth-walled, apiosporic, composed of a large curved upper cell and small lower cell, fusiform or slightly curved in shape with narrowly rounded ends, uniguttulated, lacking a gelatinose sheath, measuring (28–)29–34(–37) × (5–)6–8(–9) μm, and a basal cell 5–7 μm (*n* = 45). Asexual morph: *Mycelium* hyaline, septate, branched, hyphae 2–4 μm in diameter. *Conidiophores* reduced to the conidiogenous cells. *Conidiogenous cells* aggregated in clusters on hypha or solitary, ampuliform or cylindrical, 6–12 × 3 μm. *Conidia* brown, smooth, globose to ellipsoid (9–)10(–12) µm long (*n* = 30) in face view, lenticular, with a paler equatorial slit, and (6–)7(–8) μm long (*n* = 40) in side view. *Sterile cells* elongated, rolled up, sometimes mixed among conidia. *Culture characteristics*: ascospores germinating on MEA 2% within 24–48 h. *Colonies* flat, spreading, with sparse aerial mycelium, pale siena with white patches.

##### Type.

Portugal. *Viana do Castelo*: Valença do Minho, on dead culms of *Arundodonax*. 10 Jan. 2018, *A. Pintos* (MA-Fungi 91732 holotype, AP10118 isotype, CBS 145137 ex-type culture).

##### Notes.

*Arthriniumibericum* belongs to the large clade around *A.sacchari*, where it shows a relation with the subclade of *A.phaeospermum*, *A.saccharicola*, and the modern species *A.serenense*, *A.camelliae-sinensis*, *A.jiangxiense*, *A.dichotomanthi*, *A.obovatum* and *A.pseudosinense*. The size of conidia is more or less similar to that of *A.camelliae-sinensis*, where these measure about 9.0–13.5 μm in frontal view, but conidiogenous cells are a bit smaller in this species, measuring about 4.0–9.5 × 3.0–6.0 μm. *Arthriniumpseudosinense* has slightly smaller asci measuring 85–100 × 15–20 µm, and ellipsoid conidia covered with a mucilaginous sheath. *Arthriniumsaccharicola* has hyphae slightly wider, about 3–5 µm. The genetic identity of *A.phaeospermum* is still dubious because of the lack of a proper type, but the lineages of this species in the work of Crous and Groenewald (2013) have slightly smaller conidiogenous cells measuring 5–10 × 3–5 μm, and a different iron-grey colour of colonies in MEA.

**Figure 9.**
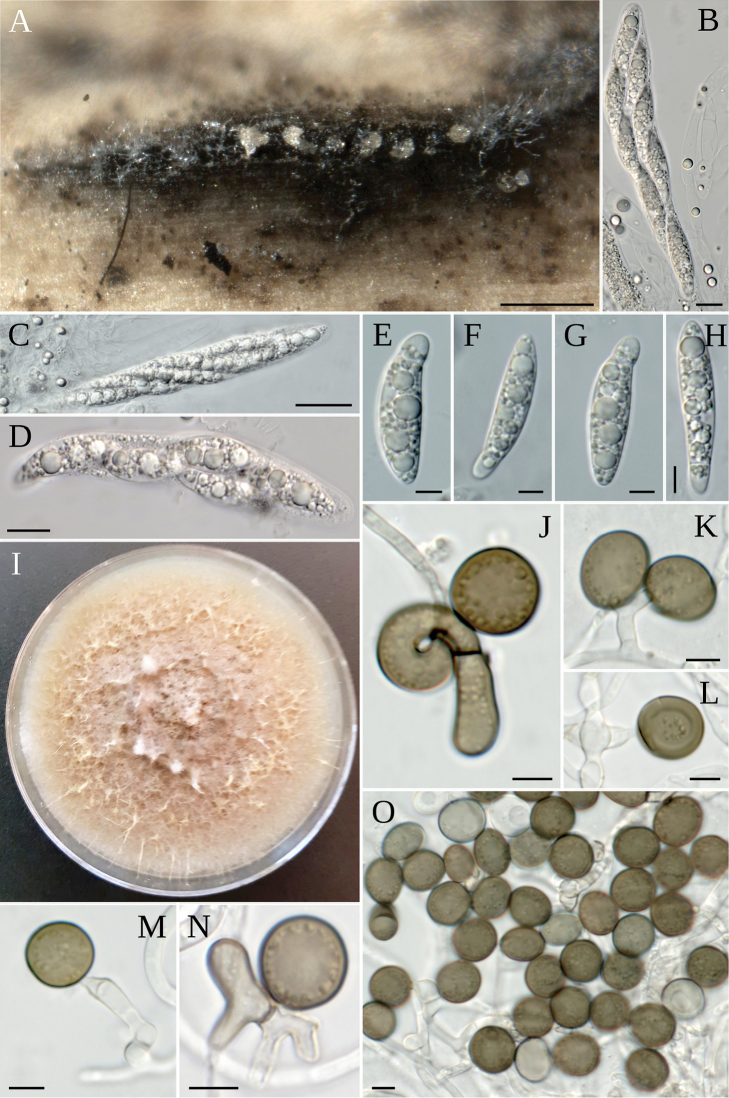
*A.ibericum***A** ascomata with oozing ascospores **B–D** asci **E–H** ascospores **I** colony on MEA**J–M** conidiogenous cells giving rise to conidia **N** sterile cell with conidia **O** conidia. Scale bars: 200 µm (**A**); 10 µm (**B–D**); 20 µm (**C**); 5 µm (**E–H**); 5 µm (**J–O**).

#### Arthrinium italicum

Taxon classificationFungiXylarialesApiosporaceae

Pintos & P. Alvarado sp. nov.

828870

Fig. 10

##### Etymology.

In reference to Italy, the country where the holotype was found.

##### Diagnosis.

Sexual morph: *Stromata* solitary to gregarious, inmersed to erumpent, fusiform, with long axis broken at the top by one or two cracks, 0.5–4 × 0.2–0.5 mm (*n* = 20). *Ascomata* uniseriate or irregularly arranged beneath stromata, pseudothecial, black, globose to subglobose with a flattened base, 150–200 μm high × 230–300 μm wide. *Peridium* composed of 5 or 6 layers of brown cells arranged in textura angularis, with a conspicuous peryphisate ostiole. *Hamathecium* paraphyses hyphae-like. *Asci* broadly cylindrical, clavate or subglobose, pedicel indistinct, apically rounded (70–)72–93(–96) × (14–)15–18(–20) μm (*n* = 30). *Ascospores* apiosporic, clavate to fusiform with narrowly rounded ends, composed of a large upper cell and small lower cell, hyaline, smooth-walled, surrounded by a gelatinose sheath, measuring (20–)21–25(–26) × (5–)6– 9(–10) μm, basal cell 3–5 μm (*n* = 45). Asexual morph: *Mycelium* consisting of smooth, hyaline, branched, septate hyphae 1.5–4 µm in diameter. *Conidiophores* straight or flexuous, cylindrical, colourless except for the thick brown transversal septa, smooth-walled, 10–50 × 1–3 μm. *Conidiogenous cells* ampuliform, cylindrical or doliform, hyaline to brown, (3–)4–7(–9) × (1.5–)2–3(–5) μm (*n* = 30). *Conidia* brown, smooth, globose in face view, lenticular in side view, 4–6 × 3–4 μm (*n* = 65), with a pale equatorial slit. *Culture characteristics*: on MEA 2%, sparse aerial mycelia, surface dirty white, reverse pale yellowish.

##### Type.

Italy: Sicily: On dead culms of *Arundodonax*, 19 June 2016, *H. Voglmayr* (MA-Fungi 91733 holotype, AP221017 isotype, CBS 145138 ex-type culture).

##### Notes.

*Arthriniumitalicum* is phylogenetically close to *A.thailandicum*, and to a lesser extent to *A.malaysianum*. Stromata of *A.thailandicum* are smaller than those of *A.italicum*, measuring 0.45–0.99 × 0.3–0.55 mm, ascomata are perithecical, its conidiogenous cells are longer (11.5–39 × 2–3.5 μm) and branched, and conidia measure 5–9 × 5–8 μm. The conidia of *A.malaysianum* are similar in size, but this species does not produce conidiophores.

##### Other specimens examined.

Spain: Balearic Islands: Mallorca, Puerto de Andratx, on dead culms of *Phragmitesaustralis*, 29 Jan. 2018, *A. Pintos* (MA-Fungi 91734, AP29118).

**Figure 10.**
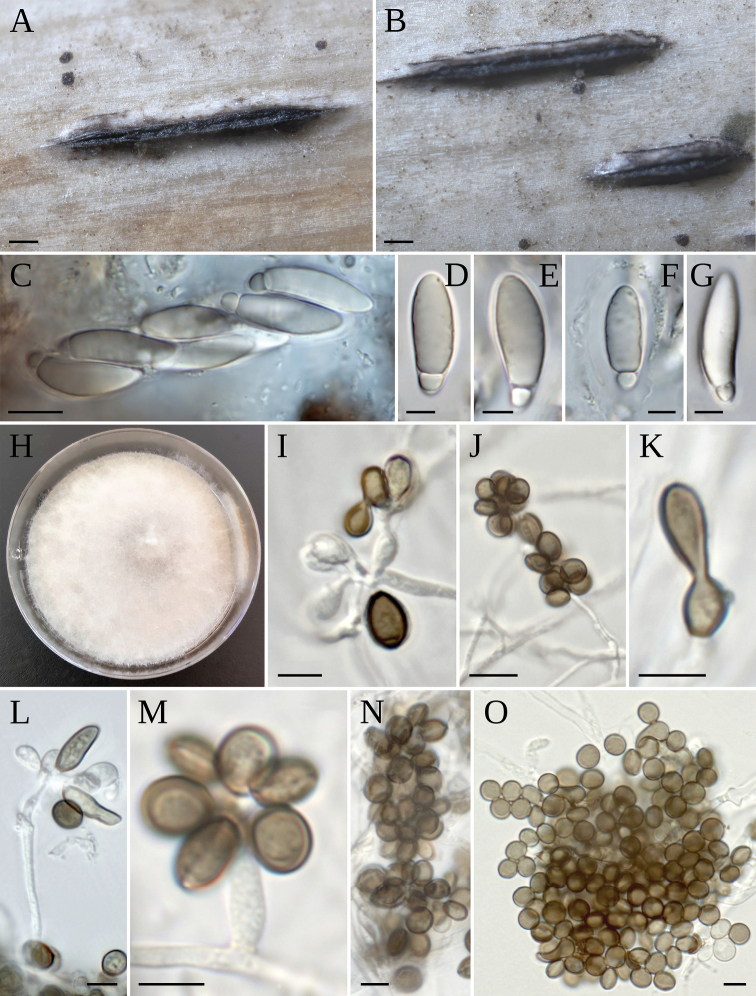
*A.italicum***A, B** stromata on host **C** asci **D, E, G** ascospores **F** ascospores with sheath **H** colony on MEA**I–M** conidiogenous cell giving rise to conidia **N, O** conidia. Scale bars: 200 µm (**A, B**); 5 µm (**D–G**); 5 µm (**H–L, N, O**); 10 µm (**M**).

#### Arthrinium marii

Taxon classificationFungiXylarialesApiosporaceae

Larrondo & Calvo, Mycologia 82: 397 (1990)

Fig. 11

##### Description.

Sexual morph: *Stromata* forming black fusiform spots, visible at the naked eye, with a long axis broken at the top revealing the ostioles of pseudothecia, 2–6 × 0.2–0.5 mm in size. *Ascomata* subglobose, sometimes with a flattened base, brownish to reddish brown, 150–190 μm high × 160–250 μm wide (*n* = 20). *Peridium* with several layers of cells arranged in *textura angularis*, with a conspicuous ostiole 50–7–80 μm diameter, periphysate. *Hamathecium* paraphyses not prominent, hyphae-like, septate, hyaline. *Asci* 8-spored, unitunicate, broadly cylindrical to clavate, with rounded apex and a short pedicel, (60–)70–100(–115) × (16–)18–20(–22) μm (*n* = 30). *Ascospores* fusiform to elliptical, with narrowly rounded ends, hyaline, with multiple guttules, surrounded by a mucilaginous sheath, (16)19–23(–24) × (6–)7–8(–10) μm, basal cell 2–5 (*n* = 30). Asexual morph: *Mycelium* consisting of smooth, hyaline, branched, septate hyphae measuring 1.5–5 µm in diameter. *Conidiophores* straight or flexuous, cylindrical, colourless except for the thick brown transverse septa, measuring 10–40 × 2–3 μm. *Conidiogenous cells* ampuliform to cylindrical, hyaline to brown, (3–)4–7(–11) × (1.4–)2–4(–5) μm (*n* = 30). *Conidia*, brown, smooth, granular, globose in face view, lenticular in side view, measuring (6–)7–8(–9) × 4–5(–6) µm, with a pale equatorial slit. *Sterile cells* elongated, brown. *Culture characteristics*: ascospores germinating on MEA 2% within 24–48 h. *Colonies* flat, spreading, with sparse aerial mycelium, reverse concolour with agA.

##### Notes.

*Arthriniummarii* was proposed by Larrondo and Calvo (1990) who described its asexual morph. This apparently frequent species has been isolated from the atmosphere, pharmaceutical excipients, home dust, and beach sand, as well as from various plant hosts (Crous 2013). In the present work the sexual morph is described for the first time. Genetically, samples identified as *A.marii* seem to represent two distinct clades (Fig. 2), with differences in tub2 and tef1 genes, but it should be further investigated with additional data before concluding if these clades should be interpreted as intraspecific variability, partially isolated lineages, or fully isolated species. Similarly, the incomplete data from the type specimens of *A.hispanicum* and *A.mediterranei* do not allow one to conclude if these apparently related species represent a single taxon or even belong to *A.marii*.

##### Specimens examined.

Austria: Oberösterreich: St. Willibald, on dead culms of *Phragmitesaustralis*, 10 July 2016, *H. Voglmayr*, (MA-Fungi 91738, AP191017).

Italy: Sicily: casa de la Monache, on dead culms of *Phragmitesaustralis*, 16 July 2016, *H. Voglmayr* (MA-Fung 91740, APVog2).

Portugal: Viana do Castelo: Valença do Minho, on dead culms of *Phragmitesaustralis*, 10 Jan. 2018, *A. Pintos* (AP10118A).

Spain: Balearic Islands: Mallorca, Esporlas, on dead culms of *Arundodonax*, 13 July 2017, *A. Pintos* (MA-Fungi 91735, AP13717). Ibidem., 29 July 2017, *A. Pintos* (AP29717). Palma de Mallorca, on *Ampelodesmosmauritanicus*, 11 July 2017, *A. Pintos* (MA-Fungi 91737, AP11717A). Palma de Mallorca, on dead culms of *Phragmitesaustralis*, 26 July 2017, *A. Pintos* (MA-Fungi 91739, AP261017).

**Figure 11.**
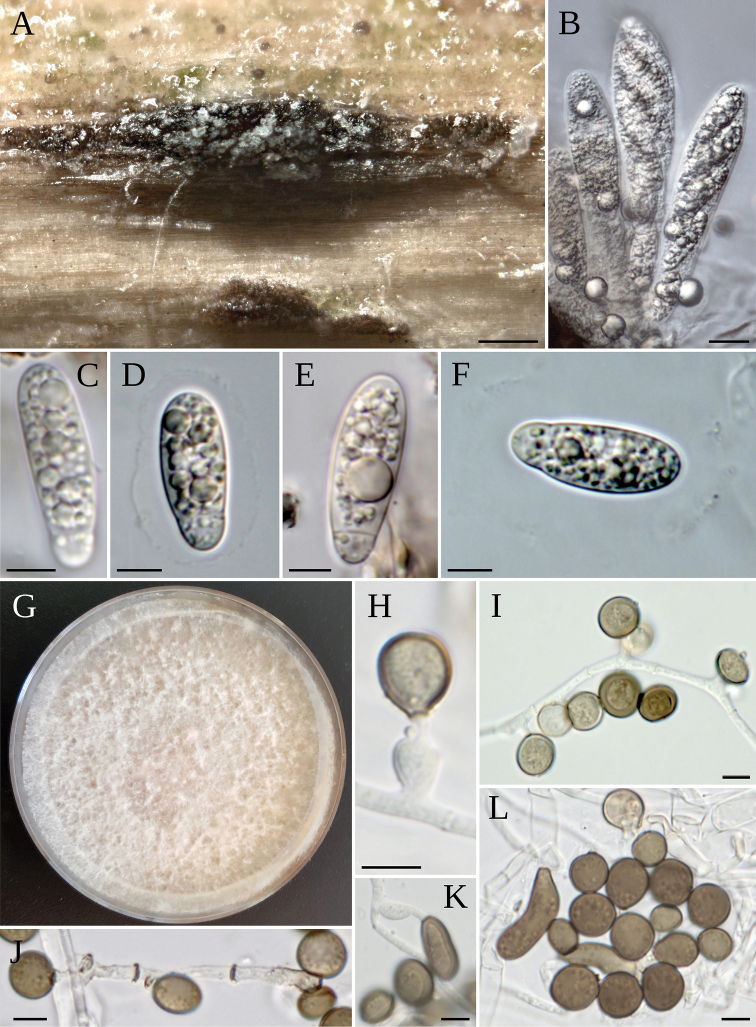
*A.marii***A** stromata on host **B** asci **C–F** ascospores **G** colony on MEA**H–I, K** conidiogenous cells giving rise to conidia **J** conidiophore bearing conidia **L** conidia and sterile cells. Scale bars: 200 µm (**A**); 10 µm (**B**); 5 µm (**C–F**); 5 µm (**H–L**).

#### Arthrinium piptatheri

Taxon classificationFungiXylarialesApiosporaceae

Pintos & P. Alvarado. sp. nov.

828871

Fig. 12

##### Etymology.

Named after *Piptatherum*, the host plant from which it was first isolated.

##### Diagnosis.

Asexual morph: *Mycelium* consisting of smooth, hyaline, branched, septate hyphae measuring 1–4 µm in diameter. *Conidiophore mother cells* hyaline to brown, aggregated in clusters or solitary on hyphae, ampuliform, cylindrical or doliform, 4–11 × 2–5 µm, growing above one or several hyaline cylindrical cells. *Conidiophore* reduced to a conidiogenous cell. *Conidiogenous cells* basauxic, polyblastic, sympodial, cylindrical, discrete, sometimes branched, smooth-walled, measuring 6–27 × 2–5 μm (*n* = 25). *Conidia* globose to ellipsoidal, pale brown to brown, with a thin hyaline germ-slit, 6–8 × 3–5 μm (*n* = 30). *Sterile cells* eloganted, brown, sometimes mixed among conidia, 13–16 × 4–5 μm (*n* = 30). *Culture characteristics*: on MEA 2%, colonies flat, spreading, with sparse aerial mycelium, reverse concolour with agar.

##### Type.

Spain: Balearic Islands: Mallorca: Llucmajor, on dead stems of *Piptatherummiliaceum*, 4 Aug. 2017, *A. Pintos* (MA-Fungi 91745 holotype, AP4817A isotype, CBS 145149 ex-type culture).

##### Notes.

*Arthriniumpiptatheri* is genetically close, but genetically distinct from *A.marii*, *A.sacchari*, *A.guizhouense*, *A.hispanicum*, *A.mediterranei*, *A.longistromum* D.Q. Dai & K.D. Hyde, and to a lesser extent *A.pseudospegazzinii* (Fig. 2) and the clade around *A.phaeospermum* (Fig. 1). The incomplete genetic data available is probably the cause behind the lack of significant support for some of these taxa. Morphologically, *A.piptatheri* differs from *A.marii* because of its sympodial, branched conidiogenous cells. *Arthriniumguizhouense* has shorter conidiogenous cells (3.5–8.0 μm). Finally, some sequences of *Ap.montagnei* are related also with this group (Fig. 2), but this species is considered the sexual morph of *A.arundinis*, with a very different genetic profile in Crous and Groenewald (2013), so its actual identity should be further investigated.

**Figure 12.**
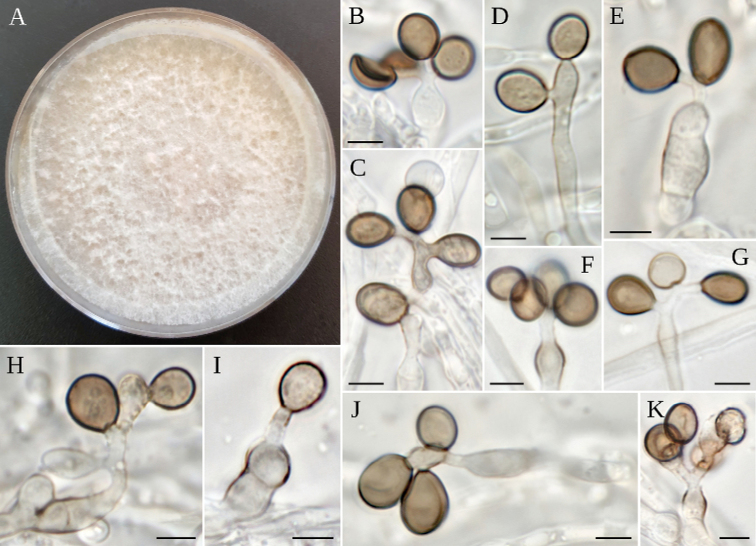
*A.piptatheri***A** colony on MEA**B–K** conidiogenous cells giving rise to conidia. Scale bars: 5 µm (**B–K**).

#### Arthrinium puccinioides

Taxon classificationFungiXylarialesApiosporaceae

Kunze & J.C. Schmidt, Mykologische (Leizpig) 2: 103 (1823)

Fig. 13

Conoplea puccinioides DE Candolle, 1905, Flore Francaise, Ed. 3, Tome 2, p.73, ex Mérat, Novuvelle Flore des environs de Paris, 1821, p. 16.
Goniosporium puccinioides (Kunze & J. C.Schmidt) Link, in Willdenow, Sp.pl., Edn 4 6(1): 44 (1824).
Gonatosporium puccinioides (Kunze & J. C.Schmidt) Corda, Icon. Fung. (Prague) 3:8 (1839).

##### Description.

Asexual morph: *Mycelium* consisting on smooth hyaline, branched, septate hyphae measuring 1.5–5 µm in diameter. *Colonies* are small, rounded or ovoid, dark brown, 50–400 µm in diameter. *Conidiophore mother cells* subspherical, lageniform or barrel-shaped, 4–5 × 3–5 µm (*n* = 30). *Conidiophores* cylindrical, straight or flexuous, septate, hyaline excepting for the thick brown or dark brown transversal septa, 20–140 × 3–4 µm (*n* = 30). *Conidiogenous cells* cylindrical, occurring between the conidiophore septa, 0.9–1.8 µm. *Conidia* dark brown, smooth, polygonal with rounded angles to hemispherical, measuring (8–)9–11(–12) × 8–9 µm, with one or two concentric pale rings. *Sterile cells* spherical, triangular or polygonal, with refractive bodies inside, paler than conidia, 6–9 µm in diameter. *Culture characteristics* colonies flat spreading on MEA 2%, with moderate aerial mycelium, reverse whitish, no esporulate on culture.

##### Notes.

*Arthriniumpuccinioides* is the only species of *Arthrinium* with polygonal conida. It shows a genetic relationship with other species found in *Carex* sp. hosts, such as *A.caricicola*, A.curvatumvar.minus, *A.japonicum* or *A.sporophleum*. The present sample fits the original description of *A.puccinioides* by Kunze and Schmidt (1823) as well as those by Ellis et al. (1951), Ellis (1965), and Scheuer (1996).

##### Specimens examined.

Germany: *Berlin*: Köpenick, Stellingdamm, on dead leaves of *Carexarenaria*, 26 April 2017, *R. Jarling* (MA-Fungi 91746, AP26418).

**Figure 13.**
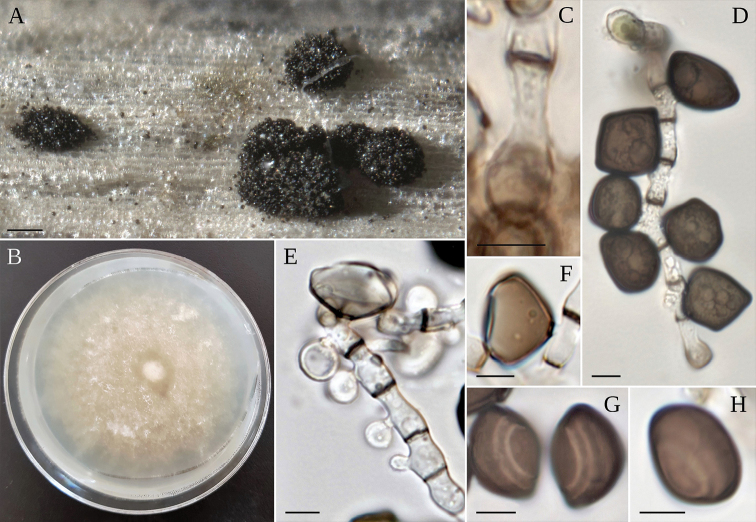
*A.puccinioides***A** colony on host **B** colony on MEA**C** conidiophore mother cell **D–F** conidiophore bearing conidia **G–H** conidia in side view. Scale bars: 100 µm (**A**); 5 µm (**C–H**).

#### Arthrinium sporophleum

Taxon classificationFungiXylarialesApiosporaceae

Kunze, 1823, in Kunze & Schmidt's Mykologische Hefte, 2, p. 104; Fries, 1832, Systema Mycol., 3, p. 377

Fig. 14

Sporophleum gramineum Nees, 1824, apud Link in Linne, Species Plantarum, ed. 4 (Willdenow's), 6, 1, p. 45.
Torula eriophori Berkeley, 1836, Fungi in J. E. Smith's English Flora, 5 (2), p. 359.
Arthrinium sporophleoides Fuckel, Jb. nassau. Ver. Naturk. 27–28: 78 (1874) [1873–74]

##### Description.

Asexual morph: *Mycelium* consisting on smooth hyaline branched hyphae, 2–5 µm in diameter. *Colonies* oval to irregular, dark blakish brown, 300–1200 × 150–650 µm. *Conidiophore mother cells* sub-cylindrical, hyaline to pale brown, measuring 5–7 × 5–7 µm (*n* = 20). *Conidiophores* straight to flexuous, cylindrical, hyaline except for the thick brown to dark brown transversal septa, 30–130 × 2–4 µm (*n* = 20). *Conidia* brown, smooth, lemon-shaped in face view, measuring (10–)11–14(–15) × (5–)6–8(–9) µm (*n* = 45), triangular with the outer edge curved and rounded angles in side view, measuring 5–8 µm thick. *Sterile cells* paler than conidia, subspherical or triangular, 5–8 µm wide. *Culture characteristics*: on MEA 2% colonies cottony, white with grey patches, reverse pale grey.

##### Notes.

*Arthriniumsporophleum* is the only species of *Arthrinium* with lemon-shaped conidia. Kunze (1823) considered that *Sporophleumgramineum* represents a synonym of this species, and Cooke (1954) considered *A.sporophleoides* Fuckel a synonym of this species too. The only sample analyzed in the present work fits the descriptions of this species by Kunze (1823), Ellis et al. (1951), Ellis (1965) and Scheuer (1996). This sample was found in *Juncus* sp., but this remarkable species has been often reported from *Carex* sp. hosts (Ellis 1965). Interestingly, other species occurring in *Carex* sp. present also conidia with unusual shapes, e.g. *A.puccinioides* (polygonal), A.curvatumvar.minus (curved), and *A.caricicola* or *A.japonicum* (fusiform).

##### Specimens examined.

Spain: Balearic Islands: Mallorca, Escorca, on dead leaves of *Juncus* sp., 21 Feb. 2018, *A. Pintos 21218* (MA-Fungi 91749).

##### Other specimens studied.

***Arthriniumarundinis***: Spain: Galicia: Santiago de Compostela, city garden, culms of *Bambusa* sp., 11 Jan. 2018, *A. Pintos 11118A* (MA-Fungi 91722). ***Arthriniumphragmitis***: Spain: Balearic Islands: Mallorca, Esporles, on dead culms of *Arundodonax*, 29 July 2017, *A. Pintos* (MA-Fungi 91744, AP29717A). Ibidem., on dead stem of *Phragmitesaustralis*, 3 Feb. 2018, *A. Pintos* (MA-Fungi 91743, AP3218). Jardin Botanico de Soller, on dead culms of *Arundodonax*, 24 Oct. 2017, *A. Pintos* (MA-Fungi 91742, AP2410172A). Puigpunyent, on dead culms of *Phragmitesaustralis*, 28 Dec. 2017, *A. Pintos* (MA-Fungi 91741, AP281217A1). ***Arthriniumrasikravindrii***: Spain: *Balearic Islands*: Mallorca, Esporlas, on dead culms of *Phyllostachysaurea*, 8 Aug. 2017, *A. Pintos* (MA-Fungi 91747, AP8817). Jardin Botanico de Soller, on dead culms of *Bambusa* sp., 24 Oct. 2017, *A. Pintos* (AP2420171). Soller, on dead culms of *Phyllostachysaurea*, 10 Apr. 2018, *A. Pintos* (MA-Fungi 91748, AP10418).

**Figure 14.**
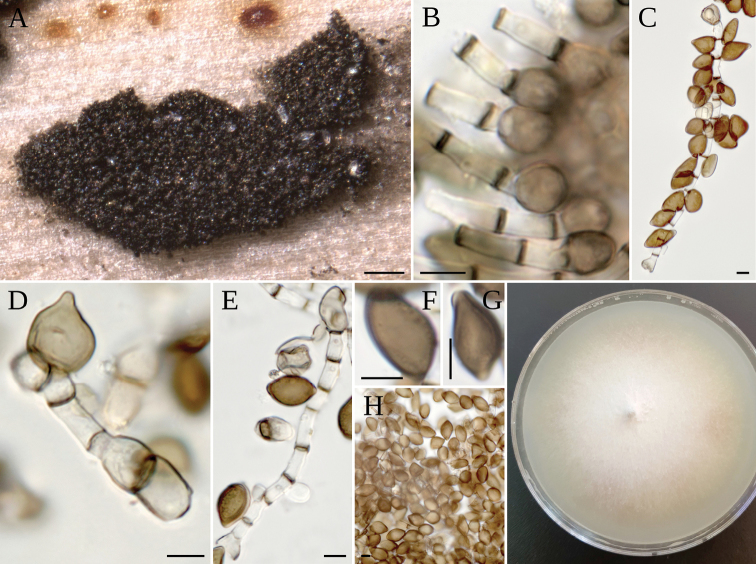
*A.sporophleum***A** colony on host **B** conidiophore mother cells **C–E** conidiophore mother cells with conidiophore bearing conidia, **F** with sterile cell **F–H** conidia **I** colony on MEA. Scale bars: 100 µm (**A**); 5 µm (**B–H**).

## Discussion

*Arthrinium* is thought to represent the asexual morph of *Apiospora* because genetic data of *Ap.montagnei* (type species of *Apiospora*, Müller and Arx 1962) grouped together with other species of *Arthrinium* (Crous and Groenewald 2013; Senanayake et al. 2015; Réblová et al. 2016). Unfortunately, no data from the type species of *Arthrinium*, *A.caricicola*, was available to confirm this synonymy. In the present work, a phylogenetic relationship was found between a specimen identified as *A.caricicola* and other species of *Arthrinium* mainly occurring in *Carex* sp., such as A.curvatumvar.minus, *A.japonicum*, *A.puccinioides* and *A.sporophleum*. Moreover, this clade was not significantly related with all other species of *Arthrinium* and *Apiospora* found in other hosts or substrates, suggesting that both clades could be interpreted as independent genera sister to *Nigrospora*. In this case, the synonymy between *Arthrinium* and *Apiospora* could be rejected, requiring new combinations. However, this hypothesis should be further confirmed after the analysis of the remaining known species occurring in Cyperaceae hosts, such as *A.austriacum*, *A.fuckelii*, *A.globosum*, *A.kamtschaticum*, *A.morthieri*, *A.muelleri*, or *A.naviculare*.

*Arthrinium* species have been found in several different plant hosts (Ramos et al. 2010; Sharma 2014), where they sometimes cause plant diseases (Martínez-Cano et al. 1992; Mavragani et al. 2007; Chen et al. 2014; Li et al. 2016). They are also isolated from lichens (He and Zhang 2012), marine algae (Suryanarayanan 2012), soil (Singh et al. 2013) and can even cause infections in humans (Rai 1989; Zhao et al. 1990; Hoog et al. 2000). In the present study six new species of *Arthrinium* are proposed: *A.balearicum*, *A.descalsii*, *A.esporlense*, *A.ibericum*, *A.italicum*, and *A.piptatheri*, all of them found in the Mediterranean biogeographical region, excepting for *A.ibericum*, which was found in the Atlantic areas of Spain. All these new taxa were found growing on plant hosts of the Poaceae family, such as *Arundodonax* or *Piptatherummiliaceum*. However, *A.marii* was the species most frequently found in the surveys, occurring on the Poaceae grasses *Ampelodesmosmauritanicus* and *Phragmitesaustralis*, in agreement with the data reported by Crous and Groenewald (2013). *Arthriniumphragmitis* was found also on *Phragmitesaustralis* and less commonly in *Arundodonax*, while *A.hysterinum* and *A.rasikravindrae* were associated with the Poaceae bamboos *Phyllostachysaurea* and *Bambusa* sp. Several colonies of *A.rasikravindrae* were found growing on *Phyllostachysaurea* as well, where they developed acervular conidiomata, a feature not observed in the protologue of this species, and therefore not considered diagnostic, in the same way as conidial shape, presence of setae, or lobate sterile cells.

*Apiosporatintinnabula* (Samuels et al. 1981) is considered a synonym of *A.hysterinum* (Sivanesan 1983; Kirk 1986). Multigenic data from the ex-type culture ICMP 6889 of *Ap.tintinnabula* was obtained so as to compare it with the newly found specimens of *A.hysterinum*, and no significant difference could be found. Interestingly, the collections of *A.hysterinum* studied in the present work presented sterile lobed cells, a feature not mentioned in the protologue of *Ap.tintinnabula*. The genetic data available from *Ap.setosa* and *Ap.bambusae* (28S and tub2) are not significantly different from those of *A.hysterinum* and *Ap.tintinnabula*, although additional markers would be needed to confirm a putative synonymy.

## Supplementary Material

XML Treatment for Arthrinium balearicum

XML Treatment for Arthrinium caricicola

XML Treatment for Arthrinium curvatum var. minus

XML Treatment for Arthrinium descalsii

XML Treatment for Arthirnium esporlense

XML Treatment for Arthrinium hysterinum

XML Treatment for Arthrinium ibericum

XML Treatment for Arthrinium italicum

XML Treatment for Arthrinium marii

XML Treatment for Arthrinium piptatheri

XML Treatment for Arthrinium puccinioides

XML Treatment for Arthrinium sporophleum
